# Das Verhältnis von Partizipation und Raum in stationären Altenhilfeeinrichtungen

**DOI:** 10.1007/s00391-023-02176-1

**Published:** 2023-04-21

**Authors:** Christian Bleck, Grit Höppner

**Affiliations:** 1 Fachbereich Sozial- und Kulturwissenschaften, Hochschule Düsseldorf, Münsterstraße 156, 40476 Düsseldorf, Deutschland; 2 Fachbereich Sozialwesen, Katholische Hochschule Nordrhein-Westfalen, Piusallee 89, 48147 Münster, Deutschland

**Keywords:** Beteiligung, Materielle Umwelt, Stationäre Altenpflege, Soziale Ungleichheiten, Involvement, Material involvement, Inpatient geriatric care, Social inequalities

## Abstract

**Hintergrund:**

Partizipation hat in der Gerontologie, Altenhilfepolitik und -praxis seit einigen Jahren Konjunktur. Dabei spielt das Verhältnis von Partizipation und Raum in der Diskussion um Quartiersorientierung eine Rolle. Wenig berücksichtigt werden bisher Zusammenhänge zwischen Partizipation und Raum innerhalb stationärer Altenhilfeeinrichtungen.

**Material und Methoden:**

Qualitative Daten aus 2 Studien zur stationären Altenhilfe werden sekundäranalytisch im Sinne einer „supra-analysis“ mittels der qualitativen Inhaltsanalyse hinsichtlich Partizipation in Bezug auf Raum und Raum in Bezug auf Partizipation untersucht.

**Ergebnisse:**

Fast alle Stufen der berücksichtigten Partizipationsleiter sind hinsichtlich der Mitgestaltung von Räumen zu finden, wobei Bewohner:innen mit Demenz weniger Partizipationsmöglichkeiten eingeräumt werden. Zudem können Räume durch ihre Anordnung Voraussetzungen für Partizipation schaffen. Wechselseitige Bezüge verdichten sich in Prozessen der Raumaneignung, Raumgestaltung und Raumplanung. Fehlen hierfür Zugänge, ist eine selbstbestimmte Alltagsgestaltung eingeschränkt.

**Diskussion:**

Die Ergebnisse tragen zu einer raumbezogenen Weiterentwicklung von Partizipationskonzepten bei, denn sie zeigen, in welchen räumlichen Zusammenhängen Partizipation in institutionellen Settings im Zusammenspiel unterschiedlicher Akteure hergestellt wird, und wie sie raumbezogen spezifisch verteilt ist. Zur Förderung von Partizipation in Einrichtungen ist eine Reflexion zu vorhandenen Möglichkeiten zur Raumaneignung, -gestaltung und -planung vor dem Hintergrund institutioneller Rahmenbedingungen bedeutend.

## Abstract (en)

**Background:**

Participation has been a key issue in gerontology, geriatric care policy and practice for several years now. The relationship between participation and space plays a role in the discussion about community orientation. So far, little attention has been paid to the relationship between participation and space within residential care facilities for older people.

**Material and methods:**

Qualitative data from two studies on residential care facilities for older people were secondarily analyzed by a “supra-analysis” and using qualitative content analysis with respect to participation in relation to space and space in relation to participation.

**Results:**

Almost all levels of the participation ladder considered can be found with respect to the co-design of spaces, although residents with dementia are granted fewer opportunities for participation. In addition, spaces can create conditions for participation through their arrangement. Reciprocal relationships are condensed in processes of space appropriation, design and planning. If there is no access to these processes, self-determined everyday life is limited.

**Conclusion:**

The results contribute to a spatially related development of participation concepts because they show in which spatial contexts participation is produced in institutional settings through the interaction of different actors and how it is distributed spatially. In order to promote participation in institutions, it is important to reflect on existing possibilities for spatial appropriation, design and planning against the background of institutional framework conditions.

Partizipation hat in der Gerontologie, Altenhilfepolitik und -praxis Konjunktur, indem der Anspruch formuliert wird, „ältere Menschen als handelnde Subjekte einzubinden“ [1]. In der stationären Altenhilfe ist dieser Bedeutungszuwachs in jüngerer Zeit durch die Stärkung der Partizipationsrechte von Menschen mit Pflege- und Unterstützungsbedarf über die UN-Behindertenrechtskonvention und (weiterentwickelte) Heimgesetze der Bundesländer begründet. Relevant ist jedoch zugleich die seit den 1970er-Jahren – oft mit Bezug auf Goffmans Konzept der totalen Institution [10] – formulierte Kritik an dem „Institutionalisierungsproblem alter Menschen im Heim“ [14], das Partizipation grundlegend verhindert [9, 19]. Das Verhältnis von Partizipation und Raum wiederum spielt in der Altenhilfe insbesondere in der Diskussion um Gemeinwesen‑, Sozialraum- bzw. Quartiersorientierung eine Rolle; hier hinsichtlich der Frage, wie partizipative Prozesse über die Öffnung von Einrichtungen für und in Quartiere angestoßen werden können [6, 7, 14, 26]. Räumlich ist der Blick damit auf Verbindungen zum Wohnumfeld gerichtet, jedoch wird das Verhältnis von Partizipation und Raum in stationären Altenhilfeeinrichtungen noch wenig berücksichtigt [21]. Dieses Verhältnis wird in diesem Beitrag – nach Erläuterung seiner theoretischen und methodischen Zugänge – untersucht.

## Theoretische Grundlagen

### Partizipation

**Figure 1:**
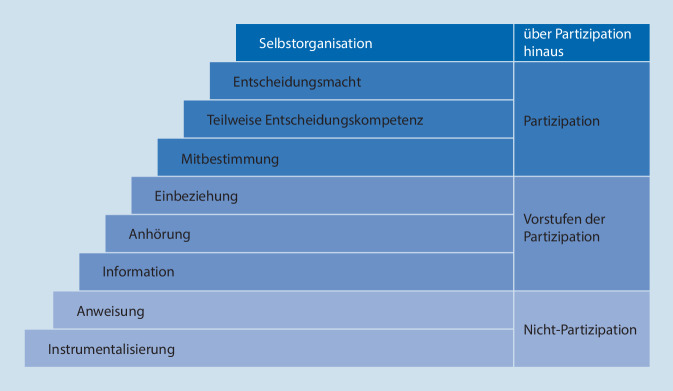

Partizipation ist ein kontextabhängiger Begriff, der in unterschiedlichen disziplinären Diskussionszusammenhängen und im deutschsprachigen Raum – im Vergleich zum englischsprachigen – mit variierenden Bedeutungen verbunden ist. Hierbei werden die Begriffe Teilhabe und Beteiligung entweder synonym oder mit Fokus auf unterschiedliche Aspekte verwendet [30]. Mit einem, beide Aspekte einschließenden Verständnis lässt sich Partizipation als „Teilhabe und Beteiligung von Menschen an gesellschaftlichen und/oder politischen Lebensbereichen“ [27] betrachten. Partizipation kann aber auch als ein spezifischer Aspekt von Teilhabe betrachtet werden: Während sich der Teilhabebegriff auf grundlegende Rechte gesellschaftlicher Zugehörigkeit bezieht, fokussiert Partizipation als Element von Teilhabe auf Bewusstseinsbildung sowie auf Voraussetzungen für Beteiligung [4]. Diese Unterscheidung ist für diesen Beitrag relevant, in dem Partizipation als besonderer Aspekt von Teilhabe mit Fokus auf die Organisation von und Mitwirkung in Entscheidungsprozessen in der stationären Altenhilfe betrachtet wird. Zudem wird berücksichtigt, dass Partizipation unterschiedliche Beteiligungsniveaus aufweisen kann [31], wofür verschiedene Stufenmodelle von Partizipation diskutiert werden [3]. Als Sensibilisierung für Beteiligungsniveaus im empirischen Material diente die in Abb. 1 aufgeführte Partizipationsleiter von Wright et al. (zit. nach [35]) – ohne zu ignorieren, dass deren lineare Stufenstruktur schematischer Natur ist.

Anregungen haben wir ferner über den Partizipationsbegriff der Lebensweltorientierung erhalten, um Partizipation in Bezug auf die individuelle Alltäglichkeit innerhalb institutionalisierter Rahmenbedingungen zu reflektieren [11].

Die Relevanz von Partizipation als demokratisches Prinzip ist auch in Kontexten des Alters unumstritten [31]. Allerdings sind Bedingungen von Partizipation, die Partizipation im Rahmen von „Altersaktivierung“ [32] als Instrument der Nutzbarmachung menschlicher Ressourcen einsetzen, um Machtverhältnisse zu konsolidieren, kritisch zu beleuchten [27]. Gleichermaßen ist auf den „Mangel an Partizipationsgerechtigkeit“ [1] hinzuweisen, und darauf, dass gerade im hohen Alter sowie bei Pflegebedarf nicht nur die individuellen Voraussetzungen für Partizipation ungleich verteilt sind, sondern auch adäquate Partizipationsstrukturen fehlen. Wenn Partizipation „auf die Ausweitung der Subjektrolle des Menschen in seiner Auseinandersetzung mit der Umwelt“ zielt [2], so sollte der Blick auch auf Zusammenhänge zwischen Partizipation und Raum gerichtet werden.

### Raum

Zur Bestimmung des Raumbegriffs, der diesem Beitrag zugrunde liegt, greifen wir zum einen auf ökogerontologische Zugänge zurück. Seit den 1970er-Jahren wurden unterschiedliche Ansätze entwickelt; diese eint, dass (Wohn)Umwelten als wesentlicher Einflussfaktor auf Altersprozesse zu verstehen sind [22, 33]. Der Begriff der Umwelt wird in frühen ökogerontologischen Ansätzen unterschieden in private Wohnumwelten wie dem Haushalt [18] und öffentliche Wohnumwelten wie institutionelle Settings [8] und Quartiere [29]. Dabei wird von einem Zusammenhang zwischen Wohlbefinden, Lebensqualität und Raum ausgegangen, und es wird die Wechselseitigkeit von Person-Umwelt-Interaktionen betont [28]. In den letzten Jahren wurden ökogerontologische Ansätze in Anlehnung an raumsoziologische Ideen weiterentwickelt, z. B. unter dem Stichwort „mapping age“ [34]. Hier wird nicht mehr nur die Wechselseitigkeit von Person-Umwelt-Interaktionen berücksichtigt, sondern die Idee der Relationalität auf den Raum selbst angewendet. Raum wird im Anschluss an Löw [23] als „relationale (An‑)Ordnung sozialer Güter und Menschen (Lebewesen) an Orten“ verstanden, wodurch die ihn konstituierenden Aushandlungsprozesse, in denen er mit Bedeutung aufgeladen wird, Aufmerksamkeit erhalten.

Zum anderen nutzen wir Überlegungen der an eine sozialraumorientierte Soziale Arbeit anschließenden Partizipativen Sozialraumforschung mit alten Menschen. Diese fokussiert weniger auf einzelne Individuen, sondern auf soziale Räume wie Quartiere sowie auf lebensweltliche Nutzungsweisen und Beziehungen der dort lebenden Menschen. Leitend ist die Frage nach den sozialräumlichen Bedingungen, die notwendig sind, um ein selbstbestimmtes Leben und soziale Teilhabe im höheren Alter mit oder ohne Pflege- und Unterstützungsbedarf zu ermöglichen [20]. Expliziter als in ökogerontologischen Ansätzen wird dabei nach den Partizipationsmöglichkeiten von vulnerablen Gruppen und sozialen Ungleichheitsverhältnissen gefragt [6, 13].

Diese Erweiterung des relationalen Raumbegriffs [16] ermöglicht es im Folgenden, das Verhältnis von Partizipation und Raum in Altenhilfeeinrichtungen empirisch auszuloten.

## Design und Methoden

Auf Basis von Daten aus 2 Studien zur stationären Altenhilfe wurde eine qualitative Sekundäranalyse im Sinne einer „supra-analysis“ [12] durchgeführt. Diese ist dadurch gekennzeichnet, dass sie über den Fokus der Primärstudie hinausgeht und deren Daten mit neuen Fragen konfrontiert. Hier handelte es sich zudem um eine nonformale Nutzung vorhandener Datensätze, bei der die Forschenden der Sekundäranalyse jeweils auch Forschende in den Primärstudien waren, deren Daten mit der folgenden Fragestellung neu analysiert wurden [25]: In welcher Weise sind Zusammenhänge zwischen Partizipation und Raum in stationären Altenhilfeeinrichtungen erkennbar?

Für die Sekundäranalyse wurde auf Transkriptionen von Expert:inneninterviews mit Einrichtungsleitungen, Leitungs- und Fachkräften der Pflege, des Sozialen Dienstes, der Hauswirtschaft und Haustechnik (*n* = 28) sowie problemzentrierten Interviews mit Bewohner:innen (*n* = 8) und Angehörigen (*n* = 12) aus der Primärstudie „Selbstbestimmt teilhaben in Altenpflegeeinrichtungen“ [5] und auf Protokolle von teilnehmenden Beobachtungen (*n* = 4) sowie auf Transkriptionen von Walking Interviews und problemzentrierten Interviews mit dem Sozialen Dienst (*n* = 5) aus der Primärstudie „Die Funktionen von Dingen in der stationären Sozialen Altenarbeit“ [15] zurückgegriffen.

Die Auswertung erfolgte über eine qualitative Inhaltsanalyse in Form der strukturierenden Analyse [24]. Der Forschungsfrage folgend wurde das Material in zwei Richtungen untersucht: Partizipation in Bezug auf Raum und Raum in Bezug auf Partizipation. Bei der analytischen Trennung dieser zwei Stränge wurden jeweils folgende Dimensionen deduktiv zur Codierung des Materials genutzt: „Aneignung von Raum“, „Anordnung von Raum“ und „Dingliche Gestaltung im Raum“. Aus forschungsökonomischen Gründen wurden drei räumliche Arrangements – „Eigene Etage und Flur“, „Veranstaltungsraum“ sowie „Garten und Terrasse“ – für die nähere Analyse unter Berücksichtigung der oben genannten sensibilisierenden Theoriezugänge ausgewählt.

## Ergebnisse

Im Folgenden werden die Ergebnisse zu Partizipation in Bezug auf Raum und Raum in Bezug auf Partizipation vorgestellt.

### Partizipation in Bezug auf Raum

Den Zusammenhang zwischen Partizipation und Raum von Partizipation ausgehend zu betrachten, bedeutet, Prozesse der partizipativen (Mit)Gestaltung von Raum durch Bewohner:innen zu analysieren.

#### Aneignung von Raum

Bewohner:innen können einen Raum über Aneignung mitgestalten. Nichtpartizipative Anweisungen der *räumlichen Verortung *verhindern jedoch Raumaneignung. So wird „auf Grundlage der (fachlichen) Meinung“ [35] entschieden, in welchen Räumen sich Teilgruppen der Bewohner:innen aufhalten: „Auf den Wohnbereichen vornehmlich die Demenzerkrankten und im Erdgeschoss-Bereich treffen sich die orientierten Bewohner“ (PDL^1^).

Raumaneignung steht auch im Zusammenhang mit *konzeptionellen Zugängen* zu Räumen. Während zeitlich flexible Angebote niedrigschwellige Zugänge und teilweise Entscheidungskompetenz in der Raumnutzung ermöglichen („[B]ei den Hühnern … gab’s … Bewohner, die jeden Tag da waren. Morgens oder abends oder mehrmals täglich“, SD), ist der Zugang zu zeitlich festgelegten Veranstaltungen hochschwelliger. Hierbei erfolgt eine Information über Aushänge oder persönliche Ansprache, und es werden viele Bewohner:innen – nach Anhörung – zum Angebot begleitet. Zudem fördern Räume mit bekannter Funktionalität Raumaneignung, indem sie alltagsnahe Raumnutzungen und Übertragungen von Entscheidungskompetenz ermöglichen, wie eine Cafeteria, in der Bewohner:innen Kuchen aussuchen können.

#### Anordnung von Raum

Mitbestimmungsmöglichkeiten der Bewohner:innen in Bezug auf die *Struktur* der räumlichen Anordnung sind nicht zu finden. So nutzt das Personal je nach Angebot auch Flure oder Essensräume und widmet diese Räume für Veranstaltungen zwischenzeitlich um. Ein Beispiel stellt ein Umbauprojekt dar, das ohne Anhörung oder Einbeziehung von Bewohner:innen geplant wurde; die Idee einer „Clubscheune“ (SD) stammt von einer Sozialarbeiter:in.

Die Anordnung von Veranstaltungsräumen hinsichtlich der *Größe* erfolgt ebenfalls durch Personalentscheidungen: „Wir räumen unheimlich viel um. Räume groß machen, Räume klein machen“ (SD).

#### Dingliche Gestaltung im Raum

Die thematisch arrangierte *Bereitstellung* von Dingen ermöglicht die Mitbestimmung von Bewohner:innen über Raumgestaltungen, etwa durch den Aufbau einer Kegelbahn für ein Angebot oder die jahreszeitliche Gestaltung eines Aufenthaltsraums: „Wir haben Tagesräume, die wir versuchen, jahreszeitlich mit zu gestalten … Wir versuchen, viel selber mit den Leuten zu machen“ (SD).

Anweisungen erfahren Bewohner:innen, wenn sie Dinge, die in spezifischen Räumen vorhanden sind, nicht allein nutzen dürfen: „Wir haben in diesem Tagesraum einen Zweiplattenherd, den ein Bewohner niemals alleine bedienen dürfte“ (SD). Diese *örtliche Nutzungsbestimmung *von Dingen, die mit einem Sicherheitsrisiko begründet wird, berücksichtigt nicht die „Einschätzung der Zielgruppe“ [35].

### Raum in Bezug auf Partizipation

Nun wird umgekehrt geprüft, inwiefern Raum Möglichkeiten der Partizipation fördert oder hemmt.

#### Aneignung von Raum

Es ist beobachtbar, dass allein das *Vorhandensein* von Räumen partizipative Raumaneignung beeinflusst. So fehlen oftmals Räume, die Bewohner:innen Selbstorganisation ermöglichen könnten. Umgekehrt bieten separat vorhandene Räume Zugänge zur Selbstorganisation, da eine Aktivität „von Mitgliedern der Zielgruppe selbst initiiert und durchgeführt“ [35] wird: „einen extra Gemeinschaftsraum …, wo die Leute von sich aus einen Spielenachmittag machen oder sich einfach so treffen“ (SD). Zudem fördern „Nischen“ (SD) als räumliche Arrangements auf den Wohnbereichen Potenziale der Selbstorganisation.

Aneignungsmöglichkeiten sind auch mit *baulichen Zugängen *verbunden. So wird die Entscheidungsmacht über Raumnutzungen gefördert, wenn Zugänge ohne Barrieren vorhanden sind: „Dieser freie Zugang in den Garten. Also Terrassentür auf, und alles kann raus und rein, wie es möchte“ (A).

Die* räumliche Lage* kann Aneignungsoptionen mindern. So schränkt eine weitere Entfernung zu Räumen die Entscheidungsmacht von Bewohner:innen ein, an dort stattfindenden Aktivitäten teilzunehmen: „die oberen Stockwerke [sind] ein bisschen vom Geschehen ab“, während „im Erdgeschoss … immer so der Trubel [ist]“ (SD).

#### Anordnung von Raum

In Bezug auf die Anordnung von Raum ist feststellbar, dass dessen* Struktur* Partizipation fördern kann. So wird ein offener, räumlicher Aufbau als niedrigeschwellige Voraussetzung partieller Entscheidungskompetenz beschrieben: „Flur ist offen gestaltet. Wenn ein Chor im Haus ist, kann er unten stehen und die Bewohner:innen können von jeder Etage aus auf den Chor blicken“ (Beobachtungsprotokoll). Demgegenüber hemmen zugebaute Räume Möglichkeiten der Bewegung und Entscheidungsmacht: „Wir haben überall Säulen, … was immer wieder störend ist“ (SD). Ferner werden Sitzanordnungen als relevant benannt, da die Sicht auf Personen und Aktivitäten Mitbestimmungsoptionen fördert.

Die *Größe *kann die Entscheidungsmacht über den Aufenthalt in Räumen beeinflussen, wenn räumliche Anordnungen zu klein bzw. eng für Bewohner:innen mit Mobilitätshilfen sind: „Was ich mir wünschen würde, wäre ein größerer Außenbereich …, es ist topografisch nicht ganz unproblematisch für Menschen, die mit Rollator oder Rollstuhl unterwegs sind“ (EL).

#### Dingliche Gestaltung im Raum

In Bezug auf Dinge ist ein *funktionales Vorhandensein* im Raum relevant, das selbstorganisierte Aktivitäten ermöglicht – wie Handläufe an Fluren für selbst initiierte Fortbewegung, Küchenzeilen und -utensilien zur eigenständigen oder begleiteten Mahlzeitenzubereitung sowie bereitgelegtes Beschäftigungsmaterial und Sitzmöglichkeiten, „wo man informell sich betätigen kann“ (SD).

Eine *örtliche Zugehörigkeit* von Dingen beeinflusst Partizipation, je nachdem wie bei Dingen, die der Institution als zugehörig bestimmt werden, Entscheidungsmacht über den Ort ihrer Nutzung gegeben ist: „Wir haben eine Leseecke, wo immer drei Zeitschriften ausliegen, wenn sie nicht wieder verlustig gehen …. Dann … können wir sie nur anbinden … mit so einem Stock. Und das wollen sie auch nicht, weil sie die gern auseinandernehmen und sich teilen“ (SD).

## Diskussion

Explorativ wurde untersucht, in welcher Weise Zusammenhänge zwischen Partizipation und Raum in stationären Altenhilfeeinrichtungen erkennbar sind. Dafür wurde eine analytische Trennung vorgenommen, durch die einerseits ersichtlich wurde, dass fast alle Stufen der Partizipationsleiter *in Bezug auf Raum* zu finden sind: Nichtpartizipative Prozesse werden durch Formen der Anweisung deutlich, indem etwa Bewohner:innen mit Demenz durch räumliche Zuweisungen in für sie vorgesehene Bereiche nicht entscheiden können, wo sie sich aufhalten. Vorstufen von Partizipation zeigen sich durch Informationen über und Anhörungen zur Teilnahme an Veranstaltungen. Partielle Übertragungen von Entscheidungskompetenz und -macht erfolgen über leicht zugängliche, flexible Angebote und die Bereitstellung von Dingen zur Raumgestaltung. Selbstorganisation ist möglich, wenn Bewohner:innen selbstinitiiert Räume „aus eigener Betroffenheit“ [35] nutzen können.

Andererseits können Einflüsse *vonseiten des Raums* den oberen und unteren, kaum aber den mittleren Stufen der Partizipationsleiter zugeordnet werden. Zu berücksichtigen ist dabei, dass eine aktive Einbeziehung in Entscheidungsprozesse kommunikative Akte verlangt, die nicht allein ausgehend von räumlichen Arrangements zu erwarten sind. Raum kann aber strukturell Entscheidungsmacht und Selbstorganisation von Bewohner:innen ermöglichen oder behindern, indem dieser vorhanden sowie gut erreichbar und niedrigschwellig nutzbar ist – oder nicht. Instrumentalisierungen und Anweisungen als Stufen der „Nichtpartizipation“ [35] erfolgen hierbei nicht situativ vom Raum ausgehend, betreffen aber im Vorfeld Prozesse der partizipativen (Mit)Gestaltung von Raum, sodass spätestens hier beide Perspektiven zusammenzudenken sind.

Werden die zwei Stränge zusammengeführt, verdichten sich wechselseitige Bezüge zwischen Partizipation und Raum in folgenden Prozessdimensionen raumbezogener Partizipation:

- *Raumaneignung* als subjektbezogene Erschließung von Raum (z. B. Entscheidungsmacht bei der Nutzung offener Räume),
- *Raumgestaltung* als dingliches Arrangieren im Raum (z. B. Entscheidungskompetenz bei der Gestaltung von Aufenthaltsräumen),
- *Raumplanung* als strukturbezogene Konzeption von Raum (z. B. Mitbestimmung bei Umbaumaßnahmen oder neuen Raumanordnungen).

**Figure 2:**
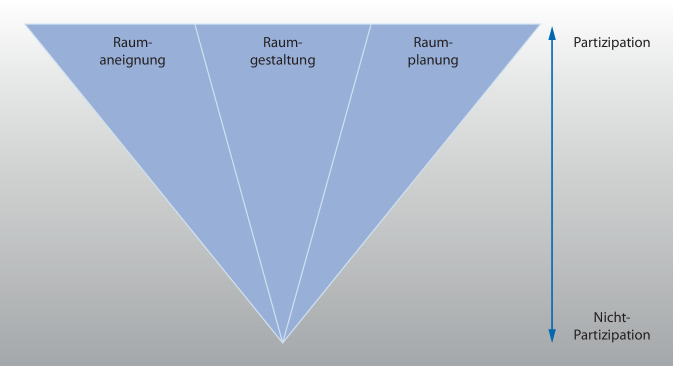

Diese drei Prozesse können also entsprechend der oben genannten Beispiele einerseits *jeweils *mehr oder minder partizipativ gestaltet werden – auf einem Kontinuum zwischen Partizipation und Nichtpartizipation. Andererseits spiegelt die Summe darauf bezogener Aktivitäten den Umfang raumbezogener Partizipation wider. Werden Raumaneignung, Raumgestaltung und Raumplanung also jeweils partizipativ ermöglicht oder umgesetzt, erhöht sich der Umfang raumbezogener Partizipation; wird hingegen einer dieser Prozesse nicht oder zu einem geringeren Anteil mit Bewohner:innen realisiert, verringert sich der Umfang (Abb. 2).

Diese Ergebnisse tragen zu einer raumbezogenen Weiterentwicklung von (Stufen)Modellen der Partizipation bei, denn sie sensibilisieren dafür, in welchen räumlichen Zusammenhängen Partizipation in – den untersuchten – institutionellen Settings im Zusammenspiel unterschiedlicher Akteure hergestellt wird, und wie sie raumbezogen jeweils spezifisch verteilt ist.

Dass Bewohner:innen mit Demenz weniger raumbezogene Partizipation als jenen ohne Demenz ermöglicht wird, ist nicht allein, aber auch als Teil des Institutionalisierungsproblems in der stationären Altenhilfe zu sehen und aus menschenrechtlicher Perspektive (z. B. auf Basis der UN-Behindertenrechtskonvention) zu problematisieren. Räumliche Anweisungen können hier nicht nur dem erhöhten Bewegungsbedürfnis von Menschen mit Demenz zuwiderlaufen, sondern ebenso zur Verräumlichung von Zuschreibungen zu Gesundheit und Krankheit beitragen. So kommen mitunter auch im Verhältnis zwischen (Nicht)Partizipation und Raum in stationären Altenhilfeeinrichtungen paternalistische Haltungen sowie damit einhergehende normierende, stigmatisierende und diskriminierende Altersbilder zum Ausdruck, die vonseiten der Kritischen Gerontologie seit Langem beanstandet werden [17].

## Schlussfolgerungen

Partizipation beschränkt sich in Altenhilfeeinrichtungen keineswegs auf die Wahl zwischen verschiedenen Menüs und Gruppenangeboten, sondern bezieht sich auch auf Prozesse der Raumaneignung, -gestaltung und -planung. Mit der selbstkritischen Reflexion und Beobachtung raumbezogener Partizipation im Alltag der stationären Altenhilfe können Fachkräfte sowohl der Verräumlichung institutionalisierter Regeln entgegenwirken als auch raumbezogene Bedürfnisse und Bedarfe von Bewohner:innen subjektorientiert unterstützen. Zugleich gilt es, diese Zugänge raumbezogener Partizipation in der Planung und Konzeption von Altenhilfeeinrichtungen zu berücksichtigen. Zur Validierung und zur Differenzierung der Erkenntnisse der vorliegenden Sekundäranalyse sind zudem Primärstudien zum Untersuchungsgegenstand, welche gezielt Daten aus Befragungen mit jenen aus Beobachtungen in Altenhilfeeinrichtungen triangulieren, zu empfehlen.

## Fazit für die Praxis

Die Ergebnisse sensibilisieren für die Bedeutung einer raumbezogenen Partizipation als Voraussetzung und Bestandteil einer selbstbestimmten Alltagsgestaltung in der stationären Altenhilfe. Um partizipative Prozesse in Einrichtungen zu fördern, erscheint eine Reflexion aufseiten der Fachkräfte zu vorhandenen Möglichkeiten der Bewohner:innen zur Raumaneignung, Raumgestaltung und Raumplanung relevant zu sein. Dies umfasst etwa Fragen der barrierearmen Nutzung von Räumen und der Bereitstellung von flexiblen Angeboten ebenso wie die der Mitbestimmung von Bewohner:innen bei der dinglichen Gestaltung und strukturellen Anordnung von Raum. Schließlich ist damit auch eine Reflexion von Verräumlichungen der Zuschreibung zu Gesundheit und Krankheit angesprochen, mittels derer institutionelle Rahmenbedingungen auf eine Festschreibung sozialer Ungleichheiten hin befragt und Regelabläufe in Altenhilfeeinrichtungen diversitätssensibel weiterentwickelt werden können.

## Footnotes

1 Abkürzungen der Interviewzitate stehen für: SD: Sozialer Dienst (inkl. Betreuungskräfte); EL: Einrichtungsleitung; PDL: Pflegedienstleitung; A: Angehörige. Zitate wurden sprachlich bereinigt und anonymisiert.
